# Editorial: Computational Biomechanics of the Heart and Vasculature With Potential Clinical and Surgical Applications

**DOI:** 10.3389/fphys.2022.872774

**Published:** 2022-04-07

**Authors:** Zhiyong Li, Youjun Liu, Estefania Peña, Daniela Valdez-Jasso, Dalin Tang

**Affiliations:** ^1^School of Mechanical, Medical and Process Engineering, Queensland University of Technology (QUT), Brisbane, QLD, Australia; ^2^School of Biological Science & Medical Engineering, Southeast University, Nanjing, China; ^3^Department of Biomedical Engineering, Faculty of Environment and Life Science, Beijing University of Technology, Beijing, China; ^4^Aragón Institute of Engineering Research (I3A), CIBER de Bioingeniería, Biomateriales y Nanomedicina (CIBER-BBN), University of Zaragoza, Zaragoza, Spain; ^5^Department of Bioengineering, University of California, San Diego, San Diego, CA, United States; ^6^Mathematical Sciences Department, Worcester Polytechnic Institute, Worcester, MA, United States

**Keywords:** biomechanics, cardiovascular disease, hemodynamics, computational modeling, numerical simulation

Mechanical forces play an important role in initiation, development, and remodeling of cardiovascular disease. Computational biomechanics has been widely applied in cardiovascular research in recent years, and extensive work has demonstrated that it has not only improved our understanding of the disease progression and remodeling following treatment, but also helped with diagnosis and treatment as well as design of medical devices. Heart and artery are active areas where computational modeling and biomechanics have made remarkable advances in investigating mechanisms governing disease development and finding optimal strategies for the management and treatment of those diseases and improving public health. Multi-disciplinary collaborations from medical imaging, bioengineering, computational modeling and clinical sciences may lead to advances in clinical applications. The goals of the Research Topic on “Computational Biomechanics of the Heart and Vasculature with Potential Clinical and Surgical Applications” were to: (a) advance ventricle biomechanical modeling to better understand ventricle function, disease initiation and development, ventricle remodeling, optimization of surgical treatment; and (b) advance vulnerable plaque biomechanical modeling to better understand mechanisms governing plaque development and rupture, plaque remodeling, optimization of surgical treatment including stenting, graft and others. Research in other areas related to cardiovascular disease was also encouraged. The Topic collects novel studies of the cross-disciplinary research in biomechanics and cardiovascular diseases. It covers a wide range of research work from disease mechanism, computational modeling, material characterization, imaging to clinical studies. Articles include new methodologies that have been recently developed to better model the hemodynamics and mechanical properties of cardiovascular system. A significant number of articles in this Research Topic are computational biomechanical simulations of the heart and vasculature with a focus on clinical and surgical applications for better patient outcomes. The second focus is using computational biomechanics to better understand the important role of hemodynamics on disease progression. There are also collections of articles demonstrating how computational modeling has contributed to medical device design and optimization. This Topic provides a comprehensive overview of the advances in biomechanical technologies and their applications in cardiovascular diseases.

## New Models and Methods

To better understand artery buckling behavior, Seddighi and Han established the theoretical foundation for non-circular blood vessel bent buckling and simulated the buckling behavior of arteries with elliptic and eccentric cross-sections using finite element analysis. Their results demonstrated that oval or eccentric cross-section increases the critical buckling pressure of arteries. To better characterize the mechanical behavior of coronary plaque, Torun et al. developed a framework of material characterization pipeline by combining *ex vivo* inflation test, inverse modeling, ultrasound/MR measurements and a machine learning-based Bayesian optimization. This could be a promising way in the future to investigate the non-linear mechanical properties of soft tissues. The importance of incorporating pre-stress in biomechanical simulation is well recognized and Fonken et al. executed time-resolved three-dimensional ultrasound-based fluid-structure interaction (FSI) simulations on an extensive set of patient data to quantify the influence of the pre-stress estimation on wall mechanics and hemodynamics. The results underline the importance of incorporating pre-stress in FSI simulations and demonstrated that their framework could provide an important tool for personalized modeling and longitudinal studies on abdominal aortic aneurysm growth and rupture risk. Moreover, considering pre-stress and anisotropy, Vignali et al. presented a new method based on a linearization strategy to perform patient- specific FSI simulations and demonstrated a significant reduction of computational costs for FSI analysis of ascending thoracic aortic aneurysm. Recent research has shown physics-based mathematical modeling and inverse method to be a useful predicting tool for hemodynamic monitoring. To verify this method in clinical setting, Bikia et al. validated a mathematical inverse-problem solving method for acquiring non-invasive estimates of mean aortic flow and stroke volume using age, weight, height and measurements of brachial blood pressure and carotid-femoral pulse wave velocity. Chen et al. established a multiscale modeling approach to reveal the vascular remodeling behavior under the interaction between the macroscale of wall shear stress (WSS) loading and the microscale of cell evolution by employing computational fluid dynamics (CFD) and agent-based model.

## Clinical Application in Diagnosis and Treatment Planning

Computational biomechanics has been widely applied in clinical applications to improve diagnosis and treatment planning. This Research Topic collects a wide arrange of such applications in different cardiovascular diseases. Coronary is one of the most active areas of research. Li et al. used 0D/3D geometric multiscale modeling method to investigate the long-term hemodynamic effects of enhanced external counterpulsation on the vascular intima in coronary artery disease treatment and demonstrated that a longer pressurization duration might result in an excessive WSS, which could easily damage the vascular intima at coronary stenosis. Banerjee et al. proposed a new hemodynamic factor - pressure drop coefficient and suggested that it could be an alternate diagnostic index for decision-making in the cardiac catheterization laboratory, in addition to fractional flow reserve. Cao et al. investigated coronary hemodynamic characteristics and suggested that patient-specific hemodynamic characteristics may play an important role which should be considered as an important factor rather than depending on coronary stenosis alone.

Aorta is another important research focus. Kan et al. applied a virtual stent-graft deployment model in patient-specific type B aortic dissection to investigate the impact of stent-graft length on wall stress distribution post thoracic endovascular aortic repair. The study demonstrated the potential of using the virtual stent-graft deployment model as a pre-surgical planning tool to help select the most appropriate stent-graft length for individual patients. Hou et al. investigated the effect of valve height on the aortic valve opening and closing and suggested that it is important to select the appropriate range of valve height for a smoother blood flow through the aortic valve and valve closure in the case of continuous aortic root dilatation in patients with aortic valve disease. Xiong et al. investigated the effects of the patent ductus arteriosus (PDA) on the flow features of the modified Blalock–Taussig shunt (MBTS) to help with preoperative surgery design and postoperative prediction. These findings suggested that preservation of PDA is not recommended during MBTS procedures because it negatively influences hemodynamics and may lead to pulmonary over-perfusion, inadequate systemic perfusion, and a heavier cardiac burden, thus increasing the risk of heart failure. Li et al. assessed the implant depth of a Venus-A prosthesis during transcatheter aortic valve replacement (TAVR) when the areas of eccentric calcification were distributed in different sections of the aortic valve. It was found that the implant depth of the Venus-A prosthesis is closely related to the distribution of eccentric calcification in the aortic valve during TAVR and suggested that future surgical strategy should consider aortic root morphology to prevent prosthesis migration.

In heart applications, Ahmed et al. performed a CFD analysis to investigate the hemodynamic effect of the endovascular Fontan revision based on anatomy, blood flow and pressure on a patient-specific model of a 2-year-old female with hypoplastic left heart syndrome. These results indicated that the proposed endovascular revision would lead to unfavorable hemodynamic conditions post procedure thereby confirming CFD modeling is a beneficial tool in surgical planning for single ventricle congenital heart defect patients. Fumagalli et al. quantified the effects of different types of hypertrophic cardiomyopathy on intraventricular blood flow and pressure gradients and suggested the computational approach is useful to guide surgical treatment of the disease.

Some other applications were also included. Tian et al. investigated of the influence of enhanced external counterpulsation (EECP) treatment on blood flow distribution and WSS-derived hemodynamic factors in the carotid bifurcation. Results indicated that EECP intervention increased internal carotid artery blood flow and WSS in the carotid bifurcation in patients with neurological disorders. Lu et al. evaluated contrast material concentration and transport in middle cerebral artery (MCA) stenosis and proposed contrast material remaining time could be a quantitative indicator to evaluate the changes in blood perfusion after the intervention for MCA stenosis. Zhang et al. quantified intracranial aneurysms hemodynamic parameters in the flow diverting stents after single- and dual-stent treatments and suggested that average pore size of stent wires at the aneurysm orifice could be a potential index for predicting the efficacy of flow-diversion treatments. Wang et al. investigated the influences of the anatomorphological features of the portal venous system on hemodynamic characteristics before and after splenectomy, with emphasis on identifying the specific anatomorphological features that make postoperative hemodynamic conditions more clot-promoting.

## Applications in Medical Device Design

Biomechanical application to medical device design is promising. Wu et al. applied CFD to optimize the hydraulic and hemolytic performance of an inhouse centrifugal maglev blood pump with a secondary flow path through variation of major design variables, with a focus on bringing down intensity of turbulence and secondary flows. The results shed light on the impact of major design variables on the performance of modern centrifugal blood pumps with a secondary flow path. In a second article, Wu et al. reported a novel design of a wearable and portable extracorporeal centrifugal blood pump based on an in-house centrifugal maglev blood pump. Compared with the baseline pump, the hydrodynamic and hemolytic performance of the portable pump was maintained without serious degradation. Li et al. explored changes in the hemodynamic behavior of the flush flow with respect to the flow injection speed and the system design to aid the design of a novel flow-blockage-free intravascular endoscope using three candidate endoscope designs. The results suggested that the endoscope design with a diameter narrowing of 30% at the endoscope neck might yield images of a better quality. Qi et al. presented the evaluation on a novel design of balloon post-dilation catheter using an experimentally verified finite element method, by comparing a newly designed spherical-tip catheter with a traditional conical-tip catheter. The study indicated that the finite element model could be a helpful tool for future optimization and evaluation of novel catheters, so as to save time and budget in product development and reduce/replace animal studies.

## Understanding Disease Progression

Computational models have also been applied to investigate cardiovascular disease progression. In aortic diseases, Pagoulatou et al. investigated acute and long-term effects of proximal aortic compliance decrease on central hemodynamics by leveraging a computational model of the cardiovascular system and demonstrated that the main mechanism that drives hypertension acutely after banding is enhancing the forward wave, which becomes even more significant after the heart remodels itself to match the increased afterload. Wang et al. conducted a quantitative CFD simulation to elucidate the local hemodynamic effects of a short-term left ventricular assist device in a patient-specific aorta model. The study provided a strategy for further predicting and assessing the effects of these hemodynamic signals on the aorta. Yan et al. compared the differences in hemodynamics and mechanical properties of bicuspid aortic valve (BAV) with different phenotypes throughout the cardiac cycle by FSI modeling. It demonstrated that the hemodynamic parameters may be critical for prediction of other subsequent aortic diseases and differential treatment strategy for certain BAV phenotype.

Deng et al. determined the mechanical effects in the formation of apical aneurysm by comparing the myofiber stress on the apical wall between healthy, subaortic obstruction, and midventricular obstruction models. The results suggested that the midventricular obstruction significantly increase the myofiber stress in the apex of left ventricle, which might directly initiate the apical aneurysm. Ghasemi et al. proposed a local stress modulated remodeling algorithm to explore the mechanical response of arterial tissue to the remodeling of collagen fibers and to understand the collagen fiber patterns required in carotid artery and plaque tissue to maintain plaque stability. Using the remodeling algorithm, the optimum fiber patterns in various patient specific plaques are identified. Wang et al. attempted to quantify patient-specific *in vivo* coronary plaque material properties based on IVUS-based multi-patient study. It was found that there is a large inter-patient and intra-patient variability in the *in vivo* plaque material properties. Wang at al. investigated the effect of flow alterations on endothelial cell distribution in the presence of gap between two struts within the parallel flow chamber. It was found that flow disturbance related to the presence of the gap and the strut orientation angle might affect the reendothelialization process on the side surfaces of the strut between the gap areas. Qing et al. analyzed the hemodynamic changes in endovascular aneurysm repair stent grafts under different configurations, angular directions, and angles. It was found that the use of the cross-limbed technique did not increase the risk of thrombosis under poor neck anatomy and the main factor affecting the hemodynamic index was the angle of the aneurysm neck, which suggested that the hemodynamic changes caused by the angle of the aneurysm neck could not be ignored when investigating the effect of the morphology of stent graft on hemodynamics.

This exciting collection of papers represent the recent developments in biomechanical modeling for cardiovascular diseases with state-of-the-art contributions on the new advances in this growing field, with an emphasis on the interface between engineering and clinical medicine. With the growing clinical needs for more accurate diagnosis of intravascular lesions, computational tools have promising potential in the field of interventional planning. The articles published in this Topic help readers understand the great potential of biomechanical simulations in improving the diagnosis and treatment of heart and vasculature diseases and create more research opportunities for clinical translational research. We are delighted to be a part of this effort and witness the rapid expanding of biomechanics in clinical applications, providing more opportunities for future discussions and exchanges of ideas and experiences in translating biomechanics into a wider range of applications. This Topic, undoubtedly, reveals the necessity to further investigate the biomechanics of the heart and vasculature with potential clinical and surgical applications. The articles in this Topic will help to promote research in this area which eventually will lead to advancement of better treatment and management technologies and strategies.

## Author Contributions

All authors listed have made a substantial, direct, and intellectual contribution to the work and approved it for publication.

## Funding

ZL received funding from NSFC (12172089, 11972118, and 61821002) and ARC (DP200103492 and DP200101970).

## Conflict of Interest

The authors declare that the research was conducted in the absence of any commercial or financial relationships that could be construed as a potential conflict of interest.

## Publisher's Note

All claims expressed in this article are solely those of the authors and do not necessarily represent those of their affiliated organizations, or those of the publisher, the editors and the reviewers. Any product that may be evaluated in this article, or claim that may be made by its manufacturer, is not guaranteed or endorsed by the publisher.

